# Maternal Weaning Modulates Emotional Behavior and Regulates the Gut-Brain Axis

**DOI:** 10.1038/srep21958

**Published:** 2016-02-23

**Authors:** Pamela Farshim, Gemma Walton, Bhismadev Chakrabarti, Ian Givens, Doug Saddy, Ian Kitchen, Jonathan R. Swann, Alexis Bailey

**Affiliations:** 1 School of Biosciences and Medicine, Faculty of Health and Medical Sciences, University of Surrey, Guildford, GU2 7XH, Surrey, UK; 2 School of Food and Nutritional Sciences, School of Chemistry, Food and Pharmacy, University of Reading, RG6 6AP, UK; 3 School of Psychology and Clinical Language Sciences, Centre for Integrative Neuroscience and Neurodynamics, University of Reading RG6 6AL, Reading, UK; 4 Food Production and Quality Division, School of Agriculture, Policy and Development, University of Reading, Earley Gate, Reading RG6 6AR, UK; 5 Division of Computational and Systems Medicine, Department of Surgery and Cancer, South Kensington campus, Imperial College London SW7 2AZ, UK; 6 Institute of Medical and Biomedical Education, St George’s University of London, London SW17 0RE, UK

## Abstract

Evidence shows that nutritional and environmental stress stimuli during postnatal period influence brain development and interactions between gut and brain. In this study we show that in rats, prevention of weaning from maternal milk results in depressive-like behavior, which is accompanied by changes in the gut bacteria and host metabolism. Depressive-like behavior was studied using the forced-swim test on postnatal day (PND) 25 in rats either weaned on PND 21, or left with their mother until PND 25 (non-weaned). Non-weaned rats showed an increased immobility time consistent with a depressive phenotype. Fluorescence *in situ* hybridization showed non-weaned rats to harbor significantly lowered *Clostridium histolyticum* bacterial groups but exhibit marked stress-induced increases. Metabonomic analysis of urine from these animals revealed significant differences in the metabolic profiles, with biochemical phenotypes indicative of depression in the non-weaned animals. In addition, non-weaned rats showed resistance to stress-induced modulation of oxytocin receptors in amygdala nuclei, which is indicative of passive stress-coping mechanism. We conclude that delaying weaning results in alterations to the gut microbiota and global metabolic profiles which may contribute to a depressive phenotype and raise the issue that mood disorders at early developmental ages may reflect interplay between mammalian host and resident bacteria.

Nutritional and environmental stress stimuli are key environmental variables that can influence brain development in early postnatal life^1^. The intake of specific nutrients is critical during the developmental period when the brain is still maturing after birth and remains sensitive to both internal and external environmental factors. Such early life influences can have a profound impact on brain development, structure and function and thus affect behavior. One of the most important events in the early stages of life is weaning, the removal of maternal milk and the introduction of solid diet. We have previously shown that weaning rat pups on postnatal day 21 (PND21), the standard age of weaning, compared to those remaining with their mother until PND25, acts as a stimulus for the activation of a population of delta-opioid receptors (DOPr), predominantly in somatosensory cortical brain regions^2^. We have shown that this weaning-induced development of DOPrs is dependent on the loss of dietary casein, a milk protein that can produce peptides with opioid activity, rather than stress due to maternal separation or loss of suckling^3^. Furthermore, it has been demonstrated that knockout mice lacking the DOPr gene exhibit increased anxiety- and depressive-like phenotypes^4^, indicating a protective role of DOPr on mood regulation. Collectively, these studies suggest a protective effect of weaning on emotional behavior.

Recent studies have pointed towards the central oxytocin system, which has also been shown to interact with the opioid receptor system^5^,^6^ as another emerging neural correlate of emotional behavioral changes induced by weaning. Oxytocin (OT) and its receptor (OTR) have been implicated as key regulators of anxiety, stress and social behaviors and exert potent antidepressant effects^7^. Environmental stimuli^8^, and experiences during early development (social, maternal care, stress) are known to shape the OT system possibly through epigenetic modification of the OTR^9^,^10^. These early life oxytocinergic changes are considered to influence life long reactivity to stressors^11^. Although, the effect of weaning on stress coping abilities is largely unknown and indeed the focus of this investigation, there is evidence that weaning^12^ can influence OTR levels in the ventromedial hypothalamus, which is likely to be involved in emotional behavioral regulation during early postnatal development.

The impact of the gut microbiota on human health and disease is now well recognized. Recent data has shown that through interactions within the gut-brain axis, the microbiota is able to influence brain development, function and behavior^13^,^14^. Probiotic bacteria such as *Lactobacillus* and *Bifidobacterium*, have been found to modulate stress responses and improve mood states^15^,^16^,^17^, whilst infections with gastrointestinal pathogens, such as *Campylobacter jejuni* and *Escherichia coli*, during the perinatal period have been associated with anxiety-like behavior and cognitive impairments^18^,^19^. Such host-microbiome interactions are bidirectional in nature with stress shown to affect gut microbial composition^20^,^21^. A variety of proposed mechanisms of action exist through which the microbiota can modulate signaling along the gut-brain axis including neural, endocrine and immunological routes^17^,^22^,^23^. Evidence has shown key chemical communications to exist between the gut microbiota and the brain via the production of neuroactive metabolites^24^. Metabonomics has emerged as a powerful tool to study such metabolites and illuminate the metabolic functionality of the microbiome and its interaction with the host.

Since weaning a) activates a population of DOPrs in the brain^2^ and is shown to have a protective effect on emotional behavior^4^ and b) influences the development of the central oxytocinergic system^12^ which is also known to regulate stress coping reactivity^8^ we have tested the hypothesis that weaning time may cause mood-related changes in postnatal rats. Given the prebiotic properties of milk and the emerging importance of the gut-brain axis in modulating behavior, we have studied the impact of weaning on the gut microbiota and its influence on the metabolic phenotypes of the host. As stress is also known to induce a marked effect on gut microbiota, we have sought to investigate the impact of stress induced by forced swimming, and its interaction with weaning on the host microbiome. Furthermore, due to the critical impact of experiences during early developmental age on shaping the central OTR system and the key role of OT in regulating stress coping capabilities we have also assessed whether weaning and stress affect central OTR binding during this early development period.

## Results

### Non-weaned Rats Display ‘Depressive-like’ Phenotypes

**Figure 1:**
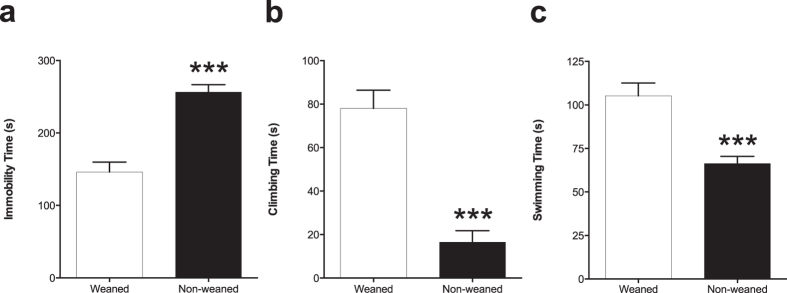
Non-weaned rats exhibit increased depressive-like behaviors. Measures were taken from (**a**) total immobility time, (**b**) time spent climbing and (**c**) in the time spent swimming from the weaned and non-weaned animals. Results show mean scores (±S.E.M) obtained from manual video analysis by three independent observers. Student’s t-test was used and a *p* value of less than 0.05 was accepted as significantly different. Non-weaned animals spent significantly more time immobile, less time to reach the first period of immobility, less time climbing and less time swimming compared to their weaned counterparts. ***p < 0.001.

We tested weaned and non-weaned rats using the forced swim test (FST) to assess depressive-like behavior. Total immobility time, swimming and climbing times as well as the number of dives were measured. Non-weaned rats showed an increase in immobility time (*P *<* *0.001; Fig. 1a). In addition, non-weaned rats spent markedly less time climbing (*P* < 0.001; Fig. 1b) and swimming (*P* < 0.001; Fig. 1c) compared to their weaned counterparts. Under normal physiological conditions animals exhibit escape-related behaviors such as diving and antidepressants are known to increase such behaviors in rats^25^,^26^. Interestingly, only the weaned rats exhibited diving activity in both pre-test and test sessions, whilst there was complete absence of this behavior in the non-weaned animals (see Supplementary Information, Movie S1 and S2).

**Figure 2:**
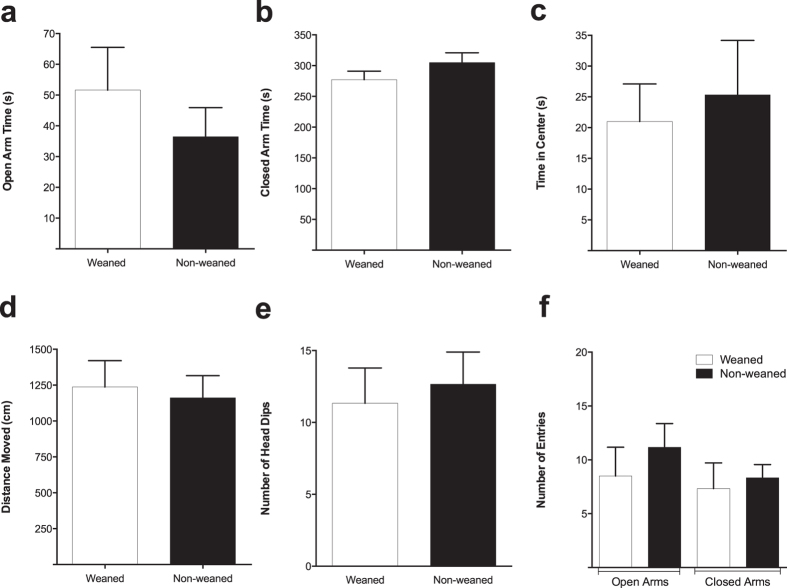
The effect of weaning on anxiety-like behaviour in the EPM. Scores are means (±SEM) of six animals per group obtained through EthoVision. Measures were taken from time in the open arms (**a**), in the closed arms (**b**) or in the time spent in the centre zones (**c**) between the weaned and non-weaned animals Measures were also taken from total distance moved (**d**), the number of entries into each arm (**e**) as well as the number of head-dips (**f**). Student’s t-test comparing weaned versus non-weaned (*p* > 0.05).

The EPM was used to assess anxiety-like behaviour in weaned and non-weaned animals. Results showed that there were no significant differences between the two animal groups in the time spent in the open arm, closed arm or in the centre zone (Fig. 2a–c). In addition, assessment of locomotor activity revealed no significant differences between the two groups in the measures obtained from the total distance moved (Fig. 2d). Furthermore, there was no significant difference in the frequency of entries into each of the arms as well as the number of head dips between the weaned and non-weaned animals (Fig. 2e,f).

### Weaning and Stress Alter Gut Bacterial Composition

Fluorescence *in situ* hybridization (FISH) analysis was used to quantify bacterial populations in the contents from five different intestinal regions of weaned and non-weaned animals in the presence or absence forced-swimming stress. Changes in the abundance of a number of bacterial groups and total bacteria were investigated using a range of oligonucleotide probes that targeted *Lactobacillus—Enterococcus* (Lab158), *Bifidobacterium* spp. (Bif164) and *Clostridium histolyticum* group (Chis150) as well as the domain bacteria probe mixture (EUBI, II & III) used for the enumeration of total bacteria.

**Figure 3:**
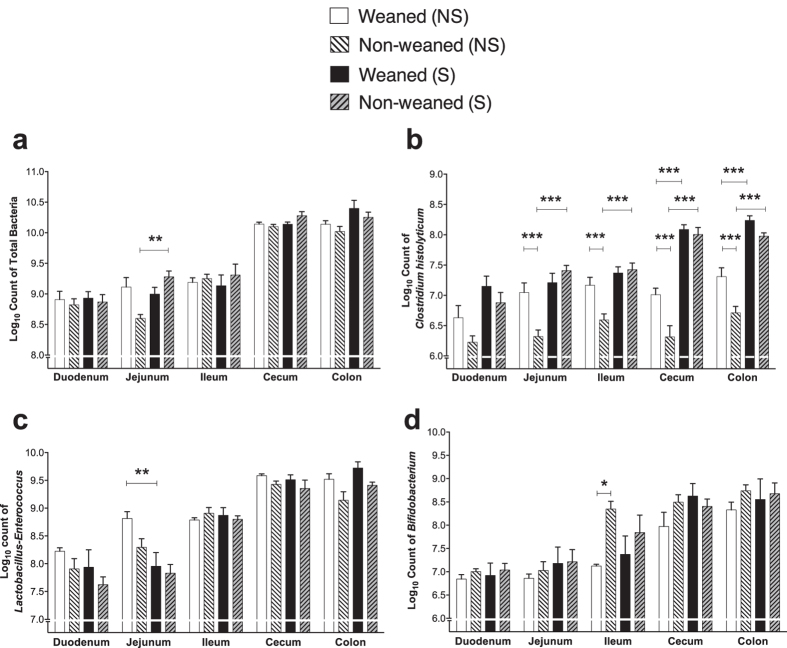
The impact of weaning and forced swim stress upon bacterial populations in intestinal contents detected by FISH using (**a**) the EUB mixture probes to enumerate total bacteria (**b**) Chis150 probe to detect bacteria belonging to the *Clostridium histolyticum* groups (**c**) Lab158 Probe for *Lactobacillus-Enterococcus* populations and (**d**) Bif164 probe to detect *Bifidobacterium* spp. levels; All data expressed as mean log_10_ cells (gram wet weight of intestinal content)^−1^, ±S.E.M (*n *=* *6 per group). Mixed modeling procedure ([‘region’], [‘weaning’], [‘region’ × ‘weaning’], [‘stress’], [‘region’ × ‘stress’], [‘weaning’ × ‘stress’] and [‘region’ × ‘weaning’ × ‘stress’]) with Bonferroni adjustments *p <* *0.05; **p <* *0.01; ***p <* *0.001. Abbreviations: NS, non-stressed; S, stressed.

Statistical analysis of the log10 of total bacterial counts (EUB) revealed a significant ‘weaning’ × ‘stress’ interaction [*F*_(1,20)_ = 4.49; *P *=* *0.0468] as well as a significant 3-way interaction between ‘region’ × ‘weaning’ × ‘stress’ [*F*_(4,17)_ = 3.94; *P *=* *0.0193] with non-weaned stressed animals harboring increased total numbers in the jejunal contents compared to their non-stressed counterparts (Fig. 3a).

Analysis of Chis150 probe log_10_ counts, showed significant overall ‘weaning’ [*F*_(1,20)_ = 14.76; *P *=* *0.001] and ‘stress’ [*F*_(1,20)_ = 92.26; *P *<* *0.0001] main effects as well as significant ‘region’ × ‘stress’ [*F*_(4,17)_ = 8.47; *P *=* *0.0006] and a ‘weaning’ × ‘stress’ [*F*_(1,20)_ = 9.08; *P *=* *0.0069] interaction effect but not a ‘region’ × ‘weaning’ × ‘stress’ interaction [*F*_(4,17)_ = 1.84; *P *=* *0.1681] (Fig. 3b). Overall, in non-weaned rats not exposed to stress, bacterial numbers were significantly lower compared to weaned animals in all but one intestinal region. Chis150 log_10_ counts were elevated in response to stress, with these increases being more pronounced in the non-weaned groups than the weaned groups (Fig. 3b).

For Lab158, there was a significant weaning [*F*_(1,20)_ = 10.11; *P *=* *0.0047] main effect and a significant ‘region’ × ‘weaning’ [*F*_(4,17)_ = 2.99; *P *=* *0.048] and ‘region’ × ‘stress’ [*F*_(4,17)_ = 4.00; *P *=* *0.0182] interactions. Stress significantly decreased Lab158 counts in the weaned but not in the non-weaned groups in the jejunum (Fig. 3c). Analysis of counts from the Bif164 probe showed a significant ‘weaning’ × ‘region’ interaction [*F*_(4,17)_ = 4.38; *P *=* *0.0129] with non-weaned rats harboring significantly greater numbers in the ileum compared to the weaned rats (Fig. 3d).

### Impact of Weaning and Stress Upon Urinary ^1^H NMR Spectroscopic Profiles

**Figure 4:**
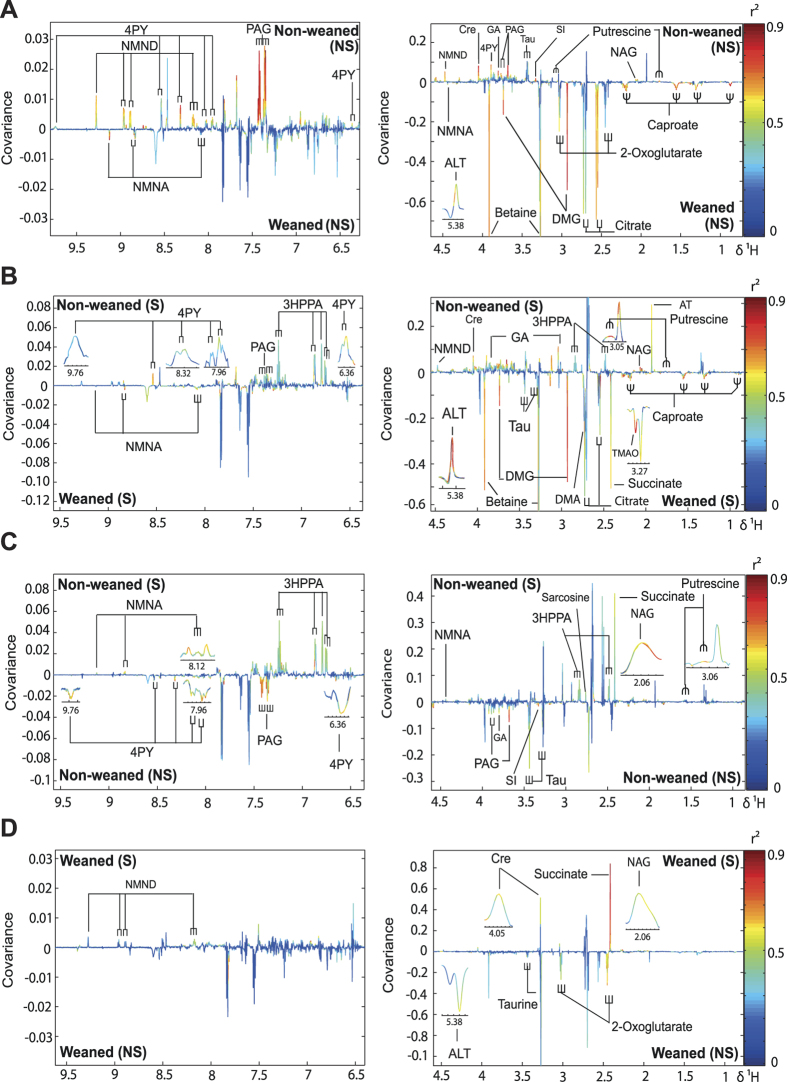
Pair-wise comparisons of urinary metabolic profiles collected from study animals using OPLS-DA. Comparing urine from (**A**) weaned and non-weaned rats (Q^2^Y = 0.82); (**B**) weaned and non-weaned rats following FST (Q^2^Y = 0.82); (**C**) non-weaned profiles with or without forced swim stress (Q^2^Y = 0.66) and (**D**) weaned animals with or without forced swim stress (Q^2^Y = 0.56). Abbreviations: 3-HPPA, 3-(3-hydroxyphenyl) propanoic acid; 4PY, *N*-methyl-4-pyridone-3-carboxamide; ALT, allantoin; AT, acetate; Cre, creatinine; DMA, dimethylamine; DMG, dimethylglycine; GA, guanidinoacetate; NAG, *N*-acetylglycoprotein; NMNA, *N*-methylnicotinic acid; NMND, *N*-methylnicotinamide; PAG, phenylacetylglycine; SI, scyllitol; Tau, taurine; TMAO, trimethylamine-*N*-oxide.

Principal Component Analysis (PCA) identified that weaning had the greatest impact on the urinary metabolic profiles resulting in higher excretion of betaine. This metabolic variation was exaggerated following exposure to forced-swimming stress (see Supplementary Fig. S1). Orthogonal Projection to Latent Structures-Discriminant Analysis (OPLS-DA) models were constructed to make pair-wise comparisons between the treatment groups (Fig. 4). Models with strong predictive ability (Q^2^Y) were returned for the comparisons between weaned and non-weaned animals in the presence or absence of forced-swimming indicating clear metabolic variation between these groups. Model validity was confirmed by permutation testing (10,000 permutations).

Comparing the urinary metabotypes from weaned and non-weaned animals (Fig. 4A) revealed that weaned animals excreted greater amounts of metabolites involved in choline metabolism such as betaine and dimethylglycine (DMG) and the energy-related metabolites citrate and 2-oxoglutarate. Non-weaned animals were found to excrete higher amounts of guanidinoacetate (GA), taurine (Tau), and *N-*acetylglycoproteins (NAG) compared to weaned rats. Variation was also observed in the excretion of microbial-derived products with non-weaned animals excreting greater phenylacetylglycine (PAG) and putrescine and the weaned animals excreting greater amounts of the medium chain fatty acid, caproate. Further variation was observed in products of nicotinic acid metabolism with *N-*methylnicotinamide (NMND) and *N*-methyl-4-pyridone-3-carboxamide (4-PY) excreted in higher amounts by the non-weaned animals and *N-*methylnicotinic acid (NMNA) excreted in higher amounts by the weaned animals (Fig. 4A). Comparing the weaned animals in the presence or absence of forced-swim stress revealed that NMND, creatinine (Cre), succinate and *N*-acetylglycoprotein excretion was increased following stress while taurine, allantoin (ALT) and 2-oxoglutarate excretion was reduced (Fig. 4D).

**Figure 5:**
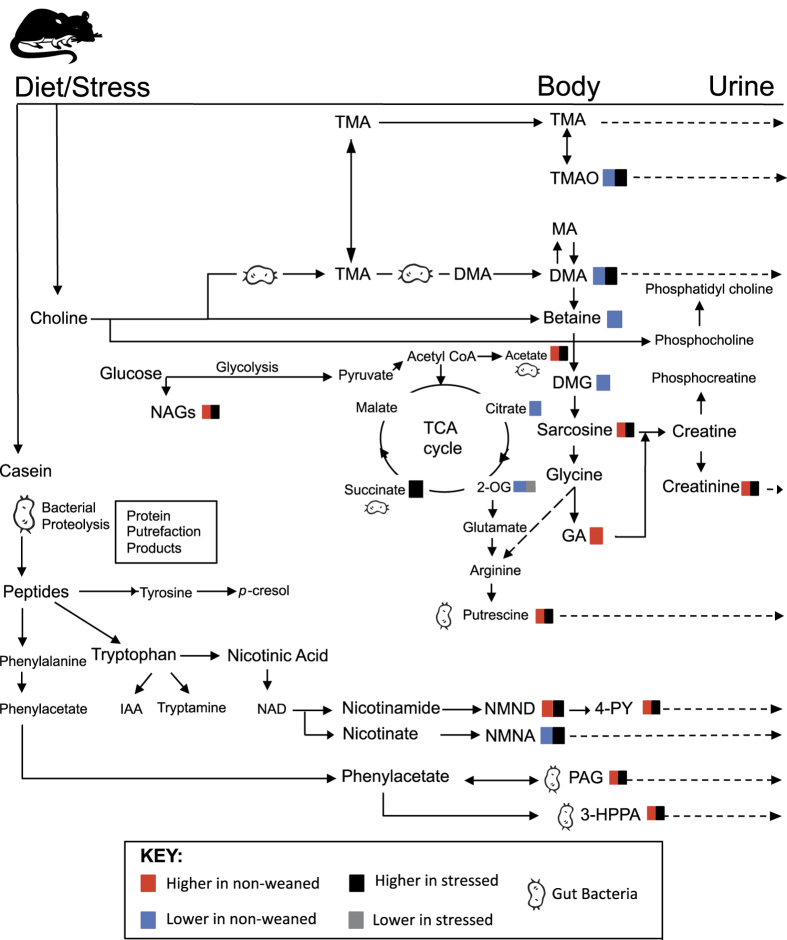
Schematic diagram illustrating the summary of metabolic pathways influenced by weaning and stress. Abbreviations: IAA, indole acetate; NAD, nicotinamide adenine dinucleotide. For other abbreviations see Table 1.

In the non-weaned animals, forced swim stress increased the urinary excretion of NMNA, 4-PY, 3-hydroxyphenylpropionic acid (3-HPPA), succinate, sarcosine, putrescine, taurine and *N-*acetylglycoproteins (Fig. 4B). Stress was also found to decrease the urinary abundance of PAG, guanidinoacetate and scyllitol (SI) in the non-weaned animals. Following the forced-swim stress the majority of weaning-associated metabolic variation persisted with the exception of 2-oxoglutarate and NMND excretion (Fig. 4D). Additional metabolic variation was observed between the weaned and non-weaned rats exposed to forced-swimming with the urine of non-weaned animals containing less succinate, TMAO, DMA and taurine than the weaned animals and greater 3-HPPA and acetate (AT) (Fig. 4B). A Summary of significant urinary metabolite changes associated with alterations in weaning and stress conditions has been shown on Table 1 and a schematic diagram shown in Fig. 5.

### Impact of Weaning and Stress upon Oxytocin Receptor Binding

**Figure 6:**
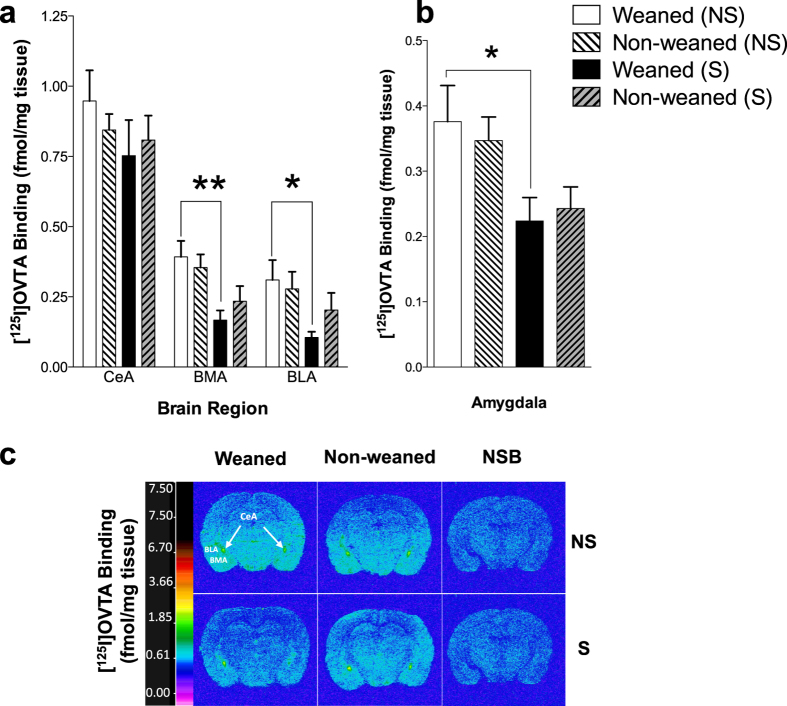
Effect of weaning and forced swim stress on oxytocin receptor (OTR) binding in the amygdala. Quantitative autoradiographic binding of OTRs in the (**a**) amygdaloid nuclei and (**b**) within the whole amygdala of the weaned and non-weaned rats in the presence of absence of forced swim stress. Data expressed as the mean specific binding of [^125^I]-OVTA (fmol/mg tissue equivalent) ± SEM (*n *=* *6). Two-way ANOVA followed by Sidak’s *post-hoc* test *p <* *0.05, **p <* *0.01. (**c**) Representative computer-enhanced autoradiograms of OTR binding from coronal brain sections of weaned and non-weaned rats in the presence of absence of forced swim stress. Color bars show a pseudo-color interpretation of the relative density of images calibrated in fmol/mg tissue equivalent. Images presented are taken from the level of the amygdala; Bregma (−2.80 mm). Representative images for the non-specific binding (NSB) (50pM [^125^I]-OVTA in the presence of 50 μM unlabeled oxytocin; third column) are also shown. Abbreviations: BLA, basolateral amygdaloid nuclei; BMA, basomedial amygdaloid nuclei; CeA, central amydaloid nuclei; NS, non-stressed; S, stressed.

Quantitative autoradiographic binding of [^125^I]-OVTA in coronal brain sections of stressed and non-stressed, weaned and non-weaned animals revealed region-specific, stress-induced alterations in OTR binding levels exclusive to the amygdala. Regional two-way ANOVA for factors ‘weaning’ and ‘stress’ showed a significant ‘stress’ effect only in the basomedial and basolateral amygdaloid nuclei (ANOVA, F_[1,20]_ = 12.50, *P* < 0.01; F_[1,20]_ = 6.14, *P* < 0.05, respectively, Fig. 6a).

Within subject analysis of OTR binding in the whole amygdala but also in the BLA and BMA of weaned and non-weaned animals revealed a significant down-regulation of OTRs in the weaned but not in the non-weaned animals (*P* < 0.05, Fig. 6b). Forced-swimming significantly decreased OTR binding in weaned rats but had no significant effect in non-weaned rats (Fig. 6a–c). No significant changes in OTR binding were observed in the other regions analyzed (see Supplementary Table S1 and Supplementary Fig. S2).

## Discussion

In this study we have shown that 25 day-old rat pups that are maintained with their mother in a non-weaned state display a depressive phenotype in comparison to pups weaned at PND 21. The increase in immobility observed in non- weaned animals is highly likely to be a representation of depressive-like behavior rather than merely an effect of general activity as no difference in locomotor activity was observed between weaned and non-weaned animals in the EPM test. Our previous investigations demonstrate that non-weaned rats lack a population of DOPrs responsible for swimming-stress-induced analgesia^2^. As DOPrs are known to modulate depressive-like behavior^4^, it is likely that a dysfunction of DOPr in non-weaned rats could at least partly underlie the depressive phenotype observed in this current study. We have previously shown that the weaning-induced activation of DOPrs involved in mediating stress-induced analgesia is independent of psychological (maternal deprivation) or physiological (suckling) stimuli, and is solely dependent on the loss of dietary casein^3^, possibly due to the removal of β-casomorphins in the gut. These peptides are known to have opioid activity^27^ and have been shown to influence emotional behavior^28^. As a result, the depressive-like phenotype described in our study is highly likely to be driven by prolonged maternal milk consumption and may have important implications for the impact of the duration of maternal milk feeding practices. Indeed, preliminary data from our laboratory show that rats which were exposed to casein rich milk from PND 21 to PND25 displayed depressive-like behavior compared to rats which were provided with casein-free milk^29^. The involvement of maternal casein milk in postnatal behavioural development though warrants further exploration.

Given the critical impact of experiences during early developmental age on shaping the central OTR system and the key role of OT in emotional regulation, we hypothesized that weaning may also affect OTR density in the brain of rats. However, consistent with the results of Curley *et al.*^12^ in male mice, we did not observe any influence of weaning on OTR density, suggesting that the phenotypic alteration in depressive-like behavior evident in PND25 weaned animals is not due to developmental changes in OTR density induced by weaning. Nonetheless, we have clearly demonstrated that weaning influences stress-induced modulation of OTR density suggesting that weaning affects the central oxytocinergic system only when the animals are exposed to acute stress. In the BMA and BLA, regions involved in emotional processing^30^, forced-swim stress induced a marked down-regulation of OTRs in weaned rats which was absent in non-weaned animals. There is evidence to suggest that the high degree of plasticity in the OTR system may constitute a protective adaptive coping mechanism in response to various insults (e.g. stress, social experiences) to shift brain function and behavior^7^. Thus, given the key role of OTR in the amygdala in the modulation of emotional responses to stress^31^, it is possible that changes in the oxytocinergic stress responses induced by weaning may represent a homeostatic stress-coping mechanism that is found to be absent in non-weaned animals remaining with the dam for a longer period of time. This hypothesis is in line with current evidence demonstrating the critical impact of experiences in early development in shaping the OT system and the key role of oxytocin in regulating long life reactivity to stressors^8^. Interestingly, no change of anxiety behavior was observed, indicating that the effect of weaning is behavior specific. It is possible that the weaning effect on emotional behavior is only apparent under conditions of higher rather than lower stress (FST vs EPM).

As milk is known to have prebiotic properties^32^, we studied the abundance of *Lactobacillus—Enterococcus* and *Bifidobacterium* spp., in the intestinal contents of weaned and non-weaned animals. *Bifidobacterium*, *Lactobacillus* and the *Clostridium histolyticum* groups were chosen for analysis as they are considered key microbial groups in terms of functionality and health modulation^33^,^34^.

However, in our study, negligible variation was observed between weaned and non-weaned animals indicating that an additional four days milk exposure was insufficient to increase the abundance of these bacterial genera. In contrast, the populations of bacteria belonging to the *Clostridium histolyticum* group were significantly higher in the weaned animals compared to those not weaned. Weaning has previously been found to expand the diversity of the microbiota and promote the growth of clostridia^32^, suggesting that the differences observed in this study are due to a nutritional component. It is important to note that the *Clostridium histolyticum* group also covers some organisms associated with negative effects, such as *Clostridium perfringens*, an organism known to have a role in antibiotic associated diarrhoea^35^.

The urinary metabotypes were studied to investigate the metabolic impact of environmental factors, namely the diet (mammalian milk exposure) and the gut microbiota, on the host. Urine is the best valuable matrix for studying this relationship as it provides a measure of the environmental inputs that are absorbed from the gut environment, which can interact with the host system and subsequently be excreted. Consistent with the microbiological profiles, variation was observed in the excretion of metabolites associated with gut microbial activity between the weaned and non-weaned animals. Non-weaned rats excreted greater amounts of PAG and putrescine, indicating greater protein putrefaction by the gut microbiota of these animals, which may relate to nutritional variation between the two animal groups. The biochemical changes observed in the non-weaned animals, in addition to lower 2-oxoglutarate and citrate, have previously been reported in a rat model of depression^36^. Furthermore, the lower excretion of choline-related metabolites, betaine, and DMG in the non-weaned animals has also been observed in depressed human subjects^37^. Betaine and DMG are products of the host’s endogenous metabolism of choline. Although weaning is known to modulate the composition of the microbiota, our observations are consistent with other metabonomic studies of depression^36^,^37^,^38^ and support the association between depression and disrupted gut microbial functionality. This connection is reinforced by the comorbidity between depressive-like and anxiety disorders and irritable bowel syndrome (IBD), a disorder recognized to involve gut microbiota disruption^38^,^39^. Depression is also associated with inflammation, and non-weaned animals were found to excrete greater amounts of *N*-acetylglycoproteins compared to the weaned animals. *N*-acetylglycoproteins are biomarkers of inflammation and have been observed in rat models of depression^36^,^40^.

Weaning also perturbed the tryptophan-nicotinic acid pathway, a pathway implicated in depression. Non-weaned animals excreted higher amounts of NMND and 4PY and weaned animals excreted greater NMNA. Tryptophan can be metabolized endogenously to either serotonin or nicotinic acid or by the gut microbiota to indican^41^. In depressed patients, an increased flux through the nicotinic acid pathway and subsequent decrease in serotonin biosynthesis has been observed^42^,^43^ with lower serotonin found in the brain of depressed individuals^44^. This is consistent with theories that relate serotonergic neurotransmission defects to depression^45^. Nicotinic acid can be methylated to form NMNA while its amine form, nicotinamide, can be metabolized to NMND and 4PY. It is unclear if higher excretion of NMND and 4-PY in the non-weaned reflect increased nicotinic acid production and decreased serotonin synthesis, since weaned animals excreted higher NMNA. Prolonged casein exposure may drive increases in NMND, given that Bell and colleagues previously reported higher urinary excretion of this compound in rats fed a purified casein diet compared to those that were on standard rat-chow diets^46^. Moreover, NMND has been shown to cause dopaminergic toxicity^47^ and destroy cerebral complex 1 subunits^48^. Elevated urinary NMND, in addition to cerebral complex 1 subunit damage has been reported in depressed individuals^37^,^49^ and interestingly, a milk-free diet has been shown to reduce damage to cerebral complex 1 subunits^50^. Collectively, our study shows that non-weaned rats that had prolonged exposure to casein, display both behavioral and biochemical phenotypes characteristic of depression and that NMND may well be involved in the mechanism driving the phenotype.

Here we show that the forced swim test, used to measure depressive-like behavior, is also acting as an acute stressor and that stress induces a marked elevation in the abundance of bacteria belonging to the *Clostridium histolyticum* group, an effect modulated by weaning. Stress-induced elevations in clostridial populations has been reported previously^21^. In the present study, we demonstrated non-weaned rats, which initially harbored lower *C. histolyticum* levels, were far more sensitive to this bacterial proliferation with elevations witnessed throughout the whole of the gastrointestinal tract. It is not clear, however, if this effect is linked to the observed behavioral phenotype. Nonetheless, given that stress-induced bacterial translocation has been linked to mood disorders such as depression^21^,^51^, it would be intriguing to suggest a possible involvement to our observed behavioral phenotype. Additionally, this is consistent with observed psychiatric comorbidities with alterations in intestinal microbiota in patients suffering from irritable bowel syndrome^52^. Consistently, several gut microbial host co-metabolites were increased in response to stress and notably 3-HPPA, a metabolite derived from microbial metabolism was excreted in greater amounts by the non-weaned animals. Multiple species of anaerobic bacteria have been shown to be involved in the production of this metabolite including the *Clostridium* genus^53^.

In conclusion, we propose that gut microbial and metabolic profiles of the host may contribute to a depressive phenotype that is observed when rats remain with their mothers until PND25. Weaning at the standard age causes fewer stress-induced alterations in gut microbiome and may induce better regulatory control of OTR in response to stress, which may, at least partly, contribute to the behavioral phenotype observed. Our studies offer novel insights into the regulatory role of weaning with respect to the biochemical interplay between the mammalian host and resident bacteria, with potential implications for manifestation of mood disorders at early developmental ages.

## Materials and Methods

### Animals

Male, Wistar albino rats (Charles River UK Limited, Kent) were used in all experiments. Four groups of animals each containing six randomly assigned male pups plus a dam were maintained in litters of 6 male pup with the dam at all times. The animals were divided into ‘weaned’ and ‘non-weaned’ groups. In the weaned groups, the mother was taken out of the cage on PND 21, whilst in the non-weaned groups the mother was kept in the cage until PND 25. Both weaned and non-weaned groups were housed on a 12 hour light-dark cycle (lights on 7:00 A.M. – off 7:00 P.M.), at a constant temperature of 22 °C ± 1 °C and humidity of 45–55% and allowed free access to rat chow and water. Weaned or non-weaned groups were exposed to behavioral testing on PND25 whilst separate groups of weaned and non-weaned animals were not exposed to any behavioral testing. PND25 was chosen as a cut off for the non weaned group as rats will stop suckling at this time and maternal milk and will resort to solid consumption^54^. This weaning period was used in previous studies carried out by Kitchen *et al.*^2^, and Goody and Kitchen^3^ who showed PND21-PND25 to be a critical window for DOPr developmental activation.

Prior to cervical dislocation all groups of animals were then individually housed in metabolic cages for 24 hours to enable collection of urine and feces. Intact brains were dissected and rapidly frozen in isopentane at −20 °C for 30 seconds and then stored at −80 °C until quantitative oxytocin receptor autoradiography. The carcasses were transferred into a fume hood and duodenum, jejunum, ileum, cecum and colon were dissected. Contents from these regions were weighed (~70–200 mg) into sterile 2 ml Eppendorf tubes and kept over ice. All experiments were conducted in accordance with the protocols approved by the Home Office, UK (Animal Scientific Procedures Act, 1986) and the local University Ethics Committee.

### Behavioral Tests

#### Forced swim test

The FST was used to assess depressive-like behaviors as originally described by Porsolt *et al.*^55^. On the day of testing (PND25) the animals were brought into the testing room in their home cages and allowed to acclimatize for 1 hour. Test chambers were cleaned with diluted odor-free detergent, rinsed and dried after each animal was tested. The rats were exposed to a 15 min ‘pre-test’ swim followed 24 hours later by a 6 min ‘test’on PND 25. The pre-test has been shown to facilitate the development of immobility during the test session and increase the sensitivity for detecting behavioral effects^56^. The rats were individually placed in glass tanks containing tap water at 23 °C ± 1 °C (diameter: 170 mm × height: 270 cm; Fisher Scientific, UK). Immediately after each session, the animals were dried by the experimenter using a paper towel and then placed in a warm recovery cage (30 °C ± 1 °C) for 5 minutes, before being returned to their home-cage. The behavior of the animal was recorded by a video camera and scored by 3 independent observers who were blind to the identity of the treatment groups of animals. The following behaviors were scored: total immobility time, swimming, climbing and diving. All tests were carried out under dim light conditions (~40 lux).

#### Elevated plus maze

Separate cohort of rats weaned at PND21 and non-weaned animals (i.e. left with the dam until PND25) were tested for anxiety behavior with the use of the elevated plus maze (EPM) on PND 25. The maze (Linton Instruments, UK) was made of black polypropylene with four equal-sized lanes (50 cm length × 10 cm width) in a shape of a plus sign and was elevated off the ground by 40 cm. The EPM consists of two arms enclosed by walls with a height of 40 cm (closed arms), and two arms with no walls (open arms). All tests were carried out under dim light conditions (~40 lux).

On PND 25, weaned and non-weaned animal groups were brought into the testing room in their home cage and left undisturbed to acclimatise for 1 hour prior to the initiation of experiments. Each rat was placed in the centre of the EPM, facing the open arm and was left to explore the whole apparatus for 6 minutes. The animal’s behaviour was recorded and data acquired with the help of the Noldus EthoVision (version 3.0) automated tracking system. Following the tests, the animals were returned to their home cage.

### Fluorescence *in situ* Hybridization (FISH)

Gastrointestinal contents from weaned and non-weaned groups of rats exposed to FST and groups of rats not exposed to any behavioural testing were diluted 1:10 (w/v) in PBS (0.1 M, pH 7.0; Oxoid). Tubes were centrifuged at 2000 *g* for 2 minutes, the supernatant collected into a 2 ml Eppendorfs and the process was repeated two times with each supernatant being added to the same 2 ml tube. The resulting supernatant was centrifuged at 13000 *g* for 5 minutes and the pellet resuspended in 750 μL of PBS and then fixed using 4% (w/v) paraformaldehyde solution (2.25 ml, Sigma) for 4–8 hours at 4 °C. Following paraformaldehyde fixation, cells were washed twice in PBS by centrifugation at 13000 *g* for 5 minutes, resuspended in 600 μL PBS/ethanol mixture (1:1) and stored at −20 °C at least 4 h prior to hybridization.

Intestinal bacterial populations were assessed by FISH analysis using a collection of 5′Cy3-labelled 16S rRNA oligonucleotide probes (Sigma Aldrich, Dorset, UK). Oligonucleotide probes targeting *Bifidobacterium spp.* (5′-CCAATGTGGGGGACCTT) Bif164; *Clostridium histolyticum* group (5′-TTATGCGGTATTAATCYCCTTT), Chis150; and lactobacilli/enterococci (5′-GGTATTAGCAYCTGTTTCCA) Lab158 were used for the enumeration of members of the gut bacteria^57^. For the enumeration of total cells, the mixture probe, EUB338I/II/III, (5′-GCTGCCTCCCGTAGGAGT, 5′-GCAGCCACCCGTAGGTGT and 5′GCTGCCACCCGTAGGTGT, respectively) was used. Slides were enumerated using an EPI-fluorescence Eclipse E400 Nikon microscope (Nikon, U.K., Kingston-upon-Thames, U.K). Fifteen random microscopic fields of view were counted per assay and used to calculate the number of cells/g of original sample.

### ^1^H Nuclear Magnetic Resonance (NMR) Spectroscopy

Urine samples from the four groups of animals (weaned and non-weaned rats exposed or not exposed to FST) were collected from the metabolic cages between 10:00 and 11:00 A.M. and stored in −80 °C. Prior to analysis, urine samples were defrosted and mixed. Samples were prepared by mixing 400 μL of urine with 200 μL of phosphate buffer at pH 7.4 containing 90% D_2_O, 1 mM 3-trimethylsilyl-1-[2,2,3,3-^2^H4] propionate (TSP) as an external reference and 2 mM sodium azide as a bacteriocide. Mixtures were then transferred into 5 mm diameter NMR tubes. For each sample the ^1^H NMR spectra were acquired using a Bruker Avance III 700 MHz spectrometer (Bruker Biospin, Rheinstetten, Germany) equipped with a cryoprobe. A standard one-dimensional NMR spectrum was obtained for each sample with water peak suppression using a standard pulse sequence (recycle delay (RD)-90°-*t*_1_-90°-*t*_m_-90°-acquire free induction decay (FID)). A RD time of 2 s was used with a mixing time (*t*_m_) of 100 ms. A total of 128 scans were acquired for each spectrum after 8 dummy scans and collected in 64 K data points using a spectral width of 12 ppm and an acquisition time per scan of 3.79 s.

### ^1^H NMR Data Processing and Analysis

Urine spectra were processed using TOPSPIN 3.0 (Bruker Biospin, Rheinstetten, Germany). The spectra were manually phased, baseline corrected, and calibrated to the TSP resonance (δ 0.00). Spectra were then digitized using in-house MATLAB (Version R2012a, The Mathworks, Inc; Natwick, MA) scripts. The water regions were eliminated to avoid baseline effects arising from imperfect water saturation. Peak alignment was applied to each spectrum to adjust peak shifts due to pH differences. Each sample was normalized using the probabilistic quotient method^58^. Chemometric analyses were performed using SIMCA-P + 11 (Umetrics AB, Umeå, Sweden) and MATLAB using scripts provided by Korrigan Sciences Ltd, UK. Principle components analysis (PCA) was performed using pareto scaling. Orthogonal Projection to latent structures-discriminant analysis (OPLS-DA) models were constructed using unit variance scaling to allow pair-wise comparisons between the treatment groups. The contribution of each variable (metabolite) to class separation was visualized by generating correlation coefficient plots. Here, the color projections corresponded to the correlation of the metabolites to class discrimination (e.g. weaned or non-weaned), with red indicating a high correlation and blue indicating a low correlation. The direction and magnitude of the peaks relate to the covariance of the metabolites to the model classes. Metabolites were assigned according to their peak shifts and multiplicity using in-house and online databases such as the Biological Magnetic Resonance Data Bank (BMRB: http://www.bmrb.wisc.edu), and the Human Metabolome Database Metabolomics Toolbox (http://www.hmdb.ca) as well as existing data in the literature.

### Quantitative Oxytocin Receptor (OTR) Autoradiography

Adjacent 20 μm coronal sections from all animal groups were cut at 400 μm intervals at −21 °C using a cryostat apparatus (Zeiss Microm 505E, Hertfordshire, U.K.) and thaw-mounted onto gelatine-coated ice-cold microscope slides to define receptor binding levels from fore- to hind-brain regions. Brain slides were stored at −20 °C in airtight containers containing a layer of anhydrous calcium sulfate (Dreirite-BDH Chemicals, Dorset, U.K.) until used. OTR autoradiography was performed on brain sections from weaned and non-weaned, forced swim stressed weaned and non-forced swim stressed non-weaned animals in accordance with previously described methods^5^ with minor modifications. Briefly, sections were rinsed twice for 10 minutes in a pre-incubation buffer solution (50 mM Tris-HCl pH 7.4) at room temperature to remove endogenous oxytocin. Total binding was determined by incubating the sections with 50 pM [^125^I]-Ornithine vasotocin analog [d(CH_2_)_5_[Tyr(Me)^2^,Thr^4^,Orn^8^,[^125^I]Tyr^9^-NH_2_]-vasotocin] ([^125^I]-OVTA) (Perkin Elmer, Boston, MA) in an incubation buffer medium (50 mM Tris-HCl, 10 mM MgCl_2_, 1 mM EDTA, 0.1% w/v bovine serum albumin, 0.05% w/v bacitracin; Sigma-Aldrich, Poole, U.K., pH 7.4 at room temperature). Adjacent sections were incubated with [^125^I]-OVTA (50 pM) for 60 minutes in the presence of 50 μM of OT ligand, (Thr^4^, Gly^7^)-oxytocin (Bachem, Germany), to determine non-specific binding (NSB).

Following the radioligand binding period, slides were rinsed 3 times for 5 minutes in ice cold rinse buffer (50 mM Tris-HCl, 10 mM MgCl_2_, pH 7.4) followed by a 30 minute wash in the ice-cold rinse buffer, and a subsequent 2 second wash in ice-cold distilled water. Slides were then dried under a stream of cool air for 2 hours and stored in sealed containers with anhydrous calcium sulphate (Drierite) for 2 days. Slides were apposed to Kodak MR-1 films (Sigma-Aldrich, U.K.) in Hypercassettes with autoradiographic [^14^C] microscales of known radioactive concentration (GE Healthcare Life Sciences, Amersham, U.K.) for 3 days and developed using 50% Kodak D19 developer (Sigma-Aldrich, Poole, U.K.).

Quantitative autoradiographic analysis of all structures were carried out by reference to the rat brain atlas of Paxinos and Watson^59^ and binding was analyzed as previously described^60^, using MCID image analyzer (Image Research, Ontario, Canada). Fmol/mg tissue equivalents for receptor binding was derived from [^14^C]-microscale (GE Healthcare, U.K.) based calibrations laid down with each film after subtraction of non-specific binding images. Optical density values were quantified from autoradiographic [^14^C] microscales of known radioactive concentration and were entered with their corresponding radioactivity values into a calibration table, and the relationship between radioactivity and optical density was subsequently determined. The following structures were analyzed by sampling 5–8 times with a box tool (box size shown in parentheses): cortex (8 × 8 mm), olfactory tubercle (6 × 6 mm) and hippocampus (5 × 5 mm). All other regions were analyzed by freehand drawing of anatomical areas. Sections for all treatment groups were processed in parallel and apposed to the same film at the same time.

### Data Analysis

The behavioral data from the FST and EPM was analyzed using an independent samples two-tailed unpaired *t-*test (weaned versus non-weaned) with GraphPad Prism 6.0 for Macintosh. A *p-*value of less than 0.05 was considered significantly different. All values were expressed as ± standard error of the mean (±SEM).

For bacterial populations, differences in log_10_ of bacterial counts were analyzed separately for individual probes using a mixed modeling procedure with intestinal region as repeated measure with unstructured variance covariance matrix, and with the following effects as independent variables: ‘region’; ‘weaning’; ‘region’ × ‘weaning’; ‘stress’; ‘region’ × ‘stress’; ‘weaning’ × ‘stress’ and ‘region’ × ‘weaning’ × ‘stress’. Linearized normal distribution curves for modeling residuals were plotted in order to check that modeling assumptions were met. The Lab158 data were found particularly to result in non-normally distributed residuals. Therefore, in this case only, the square root of the bacterial counts was used as this was found to be a suitable alternative to log_10_ of bacterial counts (used for all other individual probes) for modeling the dependent variable. Bonferroni adjustments were made for the set of comparisons to be examined within each interaction. All analyses were conducted using the SAS Software Version 9.1.3 (Statistical Analysis System Institute, Cary, NC, USA). A Bonferroni-adjusted *p* value of <0.05 was accepted as statistically significant. All data were expressed as mean ± SEM.

For OTR binding sections for all treatment groups were processed in parallel and apposed to the same film at the same time. For analysis OTR binding levels, a Two-way ANOVA was performed in each individual brain region for factors ‘weaning’ and ‘stress’ followed by Sidak’s *post hoc* analysis where applicable. All statistical analyses were performed using GraphPad Prism 6 for Macintosh. All the values were expressed as mean ± SEM.

## Additional Information

**How to cite this article**: Farshim, P. *et al.* Maternal Weaning Modulates Emotional Behavior and Regulates the Gut-Brain Axis. *Sci. Rep.* **6**, 21958; doi: 10.1038/srep21958 (2016).

## Supplementary Material

Supplementary Movie S1

Supplementary Movie S2

Supplementary Information

## Figures and Tables

**Table 1:** Summary of significant urinary metabolite differences identified between the groups by OPLS-DA.

Metabolite	Shift (multiplicity)	NW vs W (Q^2^Y = 0.82)	FST NW vs FST W (Q^2^Y = 0.82)	FST NW vs NW (Q^2^Y = 0.66)	FST W vs W (Q^2^Y = 0.56)
Microbial Derived					
3-HPPA	2.48 (t), **2.84 (t)**, 6.75 (dd), 6.79 (s), 6.86 (d), 7.24 (t)	—	+0.58	+0.64	—
PAG	3.68 (s), 3.75 (d), 7.35 (d), 7.37 (t), **7.42 (t)**	+0.93	+0.60	—0.82	—
Acetate	**1.92 (s)**	—	+0.76	—	—
Putrescine	1.76 (m), **3.06 (m)**	+0.89	+0.92	+0.78	—
Succinate	**2.41 (s)**	—	−0.77	+0.70	+0.95
Allantoin	**5.4 (s)**	+0.66	+0.88	—	−0.73
Choline Metabolism					
Betaine	3.27 (s), **3.91 (s)**	−0.82	−0.75	—	—
TMAO	**3.27 (s)**	—	−0.88	—	—
DMA	**2.74 (s)**	—	−0.75	—	—
DMG	2.93 (s), **3.73 (s)**	−0.90	−0.89	—	—
Sarcosine	**2.76 (s)**, 3.6 (s)	—	—	+0.64	—
Energy Metabolism					
2-Oxoglutarate	2.44 (t), **3.02 (t)**	−0.74	—	—	−0.81
Citrate	**2.56 (d)**, 2.71 (d)	−0.79	−0.66	—	—
Creatinine	3.05 (s), **4.05 (s)**	+0.91	+0.80	—	+0.75
Guanidoacetate	**3.8 (s)**	+0.89	+0.59	−0.73	—
Nicotinic Acid Derived					
4PY	3.89 (s), 6.36 (d), 7.4 (s), 7.95 (dd), 8.02 (s), 8.32 (d), **8.54 (d)**, 9.76 (s)	+0.82	+0.80	+0.60	—
NMNA	4.44 (s), 8.1 (t), 8.85 (t), **9.12 (s)**	−0.87	−0.73	+0.74	—
NMND	4.47 (s), 8.16 (t), 8.88 (d), 8.96 (d), **9.28 (s)**	+0.79	—	—	+0.67
Other					
Caproate	0.89 (t), 1.30 (m), 1.55 (m), **2.18 (t)**	−0.79	−0.74	—	—
Taurine	3.28 (t), **3.43 (t)**	+0.66	−0.64	+0.64	−0.72
NAG	**2.06 (s)**	+0.78	+0.90	+0.72	+0.68
Scyllitol	**3.35 (s)**	+0.72	—	−0.78	—

Values are the correlation coefficients from the OPLS-DA model obtained from resonances shown in **bold**; + indicates higher excretion in the treatment group, −, higher excretion in the relevant control groups and -, absent or non-significant in the corresponding group. 3-HPPA, 3-hydroxyphenylpropionic acid; 4-PY, *N*-methyl-4-pyridone-3-carboxamide; DMA, dimethylamine; DMG, dimethylglycine; NAG, N-acetyl-glycoproteins; NMNA, *N*-methylnicotinate, NMND, *N*-methylnicotinamide; PAG, phenylacetylglycine; TMAO, trimethylamine-*N*-oxide.
