# Supplementary material for: Maternal Weaning Modulates Emotional Behavior and Regulates the Gut-Brain Axis
Source: Sci Rep. 2016 Feb 23;6:21958. doi: 10.1038/srep21958 (PMC4763306; doi:10.1038/srep21958)
Supplement: Supplementary Information [file srep21958-s3.pdf]

Maternal Weaning Modulates Emotional Behavior and Regulates the Gut-Brain Axis

Pamela Farshim (PhD)[1,2], Gemma Walton (PhD)[2], Bhismadev Chakrabarti (PhD)[3], Ian Givens (PhD)[4], Doug Saddy (PhD)[3], Ian Kitchen (PhD)[1], Jonathan Swann (PhD)[2‡] and Alexis Bailey (PhD)[1‡*]

SUPPLEMENTARY INFORMATION

SUPPLEMENTARY FIGURES

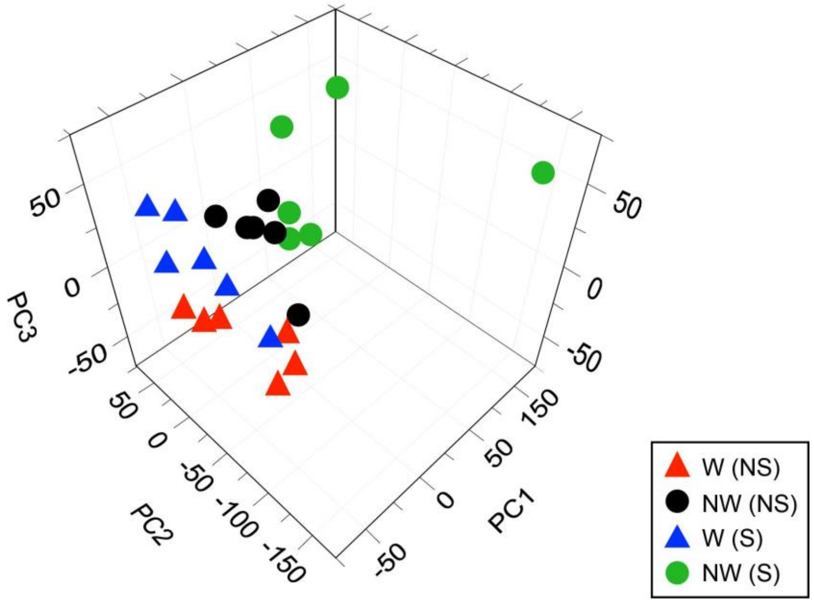

Figure S1. Principal component analysis (PCA) of the urinary metabolic profiles of all study animals. PCA scores plots comparing the urinary metabolic profiles of both weaned and non-weaned rats with and without forced swim exposure. Abbreviations: S, stressed; NS, non-stressed; W, weaned; NW, non-weaned

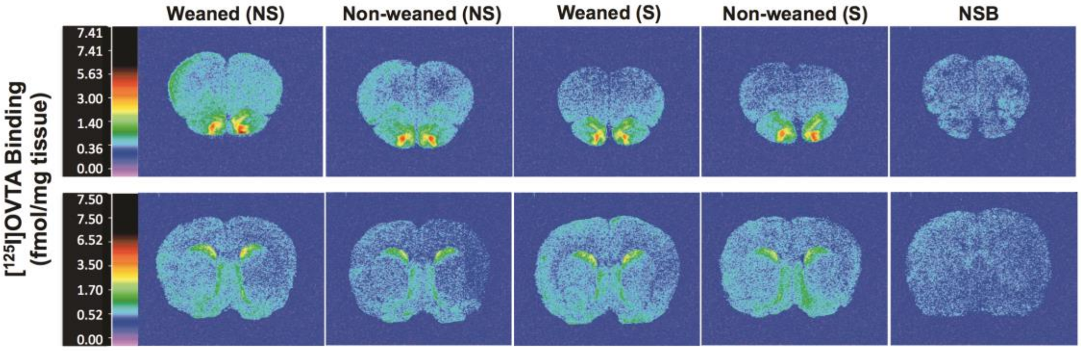

Figure S2. Quantitative OTR Autoradiography. Representative computer-enhanced color autoradiograms of OTR binding in coronal forebrain sections from weaned and non-weaned rats in the presence or absence of forced swim exposure. Color bars show a pseudo-color interpretation of the relative density of images calibrated in fmol/mg tissue equivalent. Images presented are taken from the level of the olfactory nuclei (bregma 4.20 mm, first row) and caudate (bregma 1.20 mm, second row). Representative images for the non-specific binding (NSB) (50pM [^{125}I]-OVTA in the presence of 50µM unlabeled oxytocin; third column) are also shown. OTR receptors were labeled with[^{125}I]-OVTA (50 pM) in adjacent sections from each brain. The represented images are at the level of the olfactory nuclei (bregma 4.20 mm, first row) and caudate (bregma 1.20 mm, second row). Representative images for the non-specific binding (NSB) (50pM [^{125}I]-OVTA in the presence of 50µM unlabeled oxytocin; third column) are also shown. All sections were processed in parallel throughout binding and for development of autoradiograms.

SUPPLEMENTARY TABLE 1

Quantitative autoradiography of OTR binding in weaned and non-weaned rats with and without forced swim exposure.

Brain Region	[^{125}I]-OVTA specific binding (fmol/mg tissue equivalent)			
	Weaned (NS)	Non-weaned (NS)	Weaned (S)	Non-weaned (S)
**Anterior olfactory nucleus-medial**	1.75 ± 0.20	1.67 ± 0.14	1.60 ± 0.19	1.64 ± 0.13
**Anterior olfactory nucleus-dorsal**	1.35 ± 0.17	1.40 ± 0.20	1.20 ± 0.13	1.14 ± 0.09
**Anterior olfactory nucleus-lateral**	1.44 ± 0.33	1.63 ± 0.19	1.04 ± 0.11	1.48 ± 0.20
**Anterior olfactory nucleus-ventral**	2.47 ± 0.27	2.37 ± 0.20	2.27 ± 0.45	2.62 ± 0.25
**Caudate putamen**	0.04 ± 0.01	0.06 ± 0.01	0.03 ± 0.01	0.08 ± 0.02
**Cingulate cortex**	0.92 ± 0.09	0.80 ± 0.08	0.78 ± 0.18	0.92 ± 0.07
**Bed nucleus of the stria terminalis**	0.63 ± 0.10	0.51 ± 0.09	0.48 ± 0.13	0.81 ± 0.06
**Nucleus accumbens-core**	0.17 ± 0.04	0.26 ± 0.06	0.24 ± 0.06	0.28 ± 0.04
**Nucleus accumbens-shell**	0.16 ± 0.03	0.17 ± 0.01	0.24 ± 0.07	0.25 ± 0.03
**Lateral septum**	0.40 ± 0.05	0.34 ± 0.03	0.29 ± 0.06	0.45 ± 0.03
**Ventral limp of diagonal band of Broca**	0.20 ± 0.02	0.13 ± 0.02	0.15 ± 0.03	0.23 ± 0.03
**Medial septum**	0.25 ± 0.01	0.23 ± 0.03	0.20 ± 0.04	0.30 ± 0.04
**Hippocampus**	0.09 ± 0.02	0.06 ± 0.02	0.05 ± 0.02	0.06 ± 0.02
**Thalamus**	0.06 ± 0.03	0.02 ± 0.01	0.02 ± 0.01	0.04 ± 0.01
**Hypothalamus**	0.11 ± 0.04	0.07 ± 0.01	0.06 ± 0.02	0.07 ± 0.02
**Ventromedial hypothalamic nucleus**	0.44 ± 0.09	0.39 ± 0.07	0.28 ± 0.04	0.34 ± 0.06

Data are expressed as mean specific binding (*n = 6* per group) in femtomoles per milligram (fmol/mg) ± SEM. Measures were taken at the level olfactory nuclei (bregma 4.20 mm), the caudate (bregma 1.20 mm) and thalamus (bregma -2.80 mm). Two-way ANOVA for factors ‘weaning’ and ‘stress’, followed by Sidak’s *post hoc* analysis was applied. Abbreviations: NS, non-stressed; S, stressed.

SUPPLEMENTARY MOVIES

Movie S1

The FST is a sensitive test for the assessment of depressive-like phenotypes. The 3 animals from the weaned groups in the above movie illustrates representative recording of diving behavior patterns seen in these groups during the 'test' swim session. The behavior of all animals were recorded with a video camera and later scored by three independent observers. The following behaviors were scored: immobility time, swimming, climbing and diving behavior.

Movie S2

The FST is a sensitive test for the assessment of depressive-like phenotypes. The 3 animals from the non-weaned groups in the above movie illustrates representative recording of diving behavior patterns seen in these groups during the 'test' swim session. The behavior of all animals were recorded with a video camera and later scored by three independent observers. The following behaviors were scored: Immobility time, swimming, climbing and diving behavior.
